# Inhibition of hepatic stellate cell proliferation by bone marrow mesenchymal stem cells via regulation of the cell cycle in rat

**DOI:** 10.3892/etm.2012.628

**Published:** 2012-06-29

**Authors:** SHANYU QIN, HAIXING JIANG, SIBIAO SU, DONGXU WANG, ZIYU LIANG, JUNHONG ZHANG, WEN YANG

**Affiliations:** Department of Gastroenterology, the First Affiliated Hospital of Guangxi Medical University, Nanning 530021, P.R. China

**Keywords:** bone marrow mesenchymal stem cells, stellate cells, p27, RhoA, cyclin D1

## Abstract

The present study aimed to observe the effect of rat bone marrow mesenchymal stem cells (MSCs) *in vitro* on hepatic stellate cell (HSC) RhoA signaling factors and the expression of the cell cycle regulators P27 and cyclin D1. Rat HSC-T6 and fibroblast cells were divided into control, negative control and MSC experimental groups. The cell proliferation rate was examined using the WST8 assay. The cell cycle was analyzed using flow cytometry. RT-PCR and western blot analysis were used to examine cyclin in D1 (cyclin D1), RhoA and P27 mRNA and protein expression in HSCs. After 12 h of co-culture, transition of the MSCs from the G0/G1 to S phase was blocked by HSCs. In the MSC experimental group, the *RhoA* mRNA and RhoA protein expression showed a decreasing trend with time, which was statistically significant compared with that in the control and negative control groups. MSC P27 protein expression showed an increasing trend with time. RhoA and P27 expression were significantly negatively correlated. After 24 h of co-culture, MSCs inhibited cyclin D1 expression. The difference was statistically significant in the experimental and control groups as well as in the negative control group (P<0.01). In conclusion, co-culture of HSCs with MSCs is capable of inhibiting HSC proliferation, promoting apoptosis and inhibiting RhoA expression. Reduced RhoA activity may induce an upregulation in P27 protein expression in HSCs, which promotes the inhibition of cyclin D1 by MSCs and induces cell cycle arrest at the G0/G1 phase, indicating a role in inhibiting rat HSC proliferation.

## Introduction

Various types of chronic liver fibrosis exhibit a common pathological process of liver disease. There is no specific treatment for liver fibrosis, and its end stage is liver cirrhosis. Administration of traditional treatments to attenuate the degradation process is challenging, since the mechanisms involved in liver fibrosis activate hepatic stellate cells (HSCs). Activated HSCs express α-actin (α-smooth muscle actin, α-SMA). The synthesis of large numbers of cells in the extracellular matrix (ECM) and collagen plays a key role in liver fibrosis (1). Inhibition of HSC activation or promotion of the induction of apoptosis reduces the secretion of ECM, and collagen synthesis is the key to the prevention and treatment of liver fibrosis (2). Bone marrow mesenchymal stem cells (BMSCs) are classified as being beyond non-hematopoietic stem cells in the bone marrow hematopoietic stem cells. Studies have shown that BMSCs possess differentiation potential (3–5), and that these cells may act as valuable cell sources for stem cell transplantation. Studies have also shown that MSCs effectively repair various types of liver injury, inhibit ECM deposition and reduce the degree of liver fibrosis (6,7).

In eukaryotic cells, the material bases of cell cycle regulation are the cell cycle proteins (cyclins), cyclin-dependent kinases (CDKs), cyclin-dependent kinase inhibitors (CKIs), and other intermediate factors. As a CKI member of the group, P27 plays a key regulating role in cell proliferation (8,9) and cell cycle G1/S phase transition (10). RhoA is a member of the RhoGTP kinase family, which regulates cytoskeletal dynamics, gene transcription, cell cycle progression, and cell transformation. RhoA and P27 are closely linked. RhoA activation may reduce P27 expression levels (11). MSCs co-cultured with HSCs may significantly inhibit the proliferation of the latter (12,13), although the specific mechanism is unclear. The aims of the present study were to observe the effect of MSCs on RhoA signaling factors, cell cycle protein kinase cyclin D1, and cell cycle inhibitor P27 expression, and to investigate the mechanism of MSCs in inhibiting HSC proliferation.

## Materials and methods

### Cells and animals

Six healthy Sprague-Dawley (SD) rats, 6–8 weeks old, were obtained from the Experimental Animal Center, Guangxi Medical University, Nanning, Guangxi, China. The HSC-T6 and fibroblast cell lines were purchased from the Cancer Cell Bank of the Affiliated Hospital, Sun Yat-sen University, Guangzhou, Guangdong, China.

### MSC isolation, culture, and functional identification

According to a previously used method (12), 12 SD rat femur bone marrow cells were isolated under sterile conditions, cultured at 37°C, and incubated under 5% CO_2_. The MSCs were purified by passage, and cell morphology was observed under microscopy. Passage 4 cells were digested by 2.5 g/l trypsin, and the cell concentration was adjusted to 2x10^5^/cm^2^. The cells were inoculated in 50-ml disposable culture flasks. Then, 20 μg/l (final concentration) HGF was added to the cells to induce MSCs. The solution was then cultured at 37°C and incubated under 5% CO_2_. The medium was changed once every 3 days and continuously cultured for 14 days. Cell morphology was observed under inverted phase contrast microscopy.

### HSC-T6 culture, passage, and activation identification

Rat HSC-T6 was cultured in an L-DMEM medium containing 100 ml/l fetal calf serum at 37°C in a 5% CO_2_ incubator. The cells grew at 8 h, and 80–90% of the cells adhered to the bottom of the bottle after 2–3 days for passage. The active 3rd–4th generation cells were used in the experiments. The α-SMA expression was examined using immunohistochemistry. The morphological changes of the living cells were observed under inverted phase contrast microscopy.

### Cell co-culture

According to a previously described method (14,15), the MSCs or fibroblasts were inoculated in a semi-permeable membrane (transwell insert) in the upper part (2x10^5^ cells/well) of a cell culture 6-well plastic box. The HSC-T6 cells were inoculated in the lower part (2x10^5^ cells/well) to establish the upper and lower double-cell co-culture system. The experiments were divided as follows: i) control group, HSCs cultured alone (only the upper layer contained the medium); ii) negative control group, HSCs cultured with fibro-blasts; iii) MSC experimental group, MSCs cultured with HSCs. The three groups were observed at 0, 6, 12, 24, 48, and 72 h. The dynamic morphology of the living cells was observed through an inverted phase contrast microscope.

### HSC proliferation rate

After each period of co-culture, the adherent cells were digested with 2.5 g/l trypsin. The cell concentration was adjusted to 2x10^5^ cells/ml and mixed thoroughly. Then, 100 μl of these cells was added into each well of 96-well plates, followed by the addition of 10 μl CCK-8 solution. The solution was incubated for 1 h and then examined at 450 nm. The mean value was obtained.

### Cell cycle detection

The MSCs and HSCs were co-cultured at 2x10^5^ cells/well. Cells were obtained at different intervals, and adherent cells were digested by trypsin, washed with PBS and fixed with 70% pre-cooled ethanol at 4°C overnight. An equal amount of PBS was added twice for washing. Up to 100 μl RNase A was added at 37°C for 30 min, followed by the addition of propidium iodide at 4°C in the dark for 30 min. Cell cycle was analyzed by flow cytometry using the MCYCLE software (Beckman, New York, NY, USA).

### RNA extraction and RT-PCR

The HSCs were collected and counted at each period. TRIzol was added to extract the total RNA according to the instructions in the kit. The target gene was amplified according to the following conditions: 95°C pre-denaturation for 5 min, 95°C denaturation for 45 sec, 55°C annealing for 45 sec, 72°C for 1 min for 35 cycles, and 72°C for 5 min. GAPDH was used as an internal reference. The primers used were as follows: RhoA upstream, 5′-TGGTGA TGGAGCTTGTGGTAAG-3′; downstream, 5′-AACATCAGT GTCTGGGTAGGAG-3′; P27 upstream, 5′-TGCAACCGA CGATTCTTCTACTCAA-3′; downstream, 5′-CAAGCAGTG ATGTATCTGATAAACAAGGA-3′; cyclin D1 upstream, 5′-TGTTCGTGGCCTCTAAGATG-3′; downstream, 5′-ACT CCAGAAGGGCTTCAATC-3′; and GAPDH upstream, 5′-GCCAGTAGACTCCACGACAT-3′; downstream, 5′-GCA AGTTCAACGGCACAG-3′. A total of 6 μl PCR products was examined using 1.7% agarose gel electrophoresis and scanned under a gel image analysis system to observe the gray ratio of the target gene/GAPDH, representing the target gene mRNA levels.

### Western blot analysis

The HSC total proteins from each period were extracted with cell lysate. Protein concentration was determined using the Coomassie brilliant blue colori-metric method. Proteins (80 μg) were run on 15% SDS-PAGE gel electrophoresis, transferred onto the PVDF membrane, and then blocked. Anti-mouse anti-RhoA, P27 mAb, and cyclin D1 antibody (1:500 dilution) were added sequentially and then incubated at 4°C overnight. A secondary antibody labeled by horseradish peroxidase-conjugate was added for hybridization. The solution was then incubated with ECL luminescence agent for 1–5 min, exposed, developed, and fixed. Digital image analysis software (Bio-Rad, New York, NY, USA) was used to analyze the results. The target protein/GAPDH ratio indicated the relative target protein expression level.

### Statistical analysis

Data are expressed as the means ± SD and analyzed using the statistical software SPSS13.0. P<0.05 was considered to indicate statistical significance.

## Results

### Identification of the HSC-T6 activity

The immunohisto-chemical staining results showed the positive HSC α-SMA expression following 48 h of culture. The cytoplasm was stained brown with a thin streak. The HSC was star-shaped, with a large cell body and stretched membrane. The positive rate of α-SMA was >95%.

### HSC morphological changes

The HSCs showed no significant change in morphology following co-culture with MSCs from 0 to 24 h. The cells were mostly oval with weakened membrane stretching, smaller refractive index particles were exhibited, cell adhesion decreased, and cell number decreased at 48 h. The HSCs were round or oval without membrane stretching, the refractive index particle became dense, adhesion was poor, and the cell number decreased significantly at 72 h. Following co-culture, the HSCs showed no significant morphological change between the blank and the negative control groups. The HSCs appeared as stars, with large cell bodies and membrane stretching and with low refractive-index particles.

### Detection of HSC proliferation rate

The cells in the control group were used as reference values. The MSCs caused mild inhibition at 24 h, with an inhibition rate of 5.15±2.1%. Afterward, cell proliferation inhibition was significantly enhanced, with 16.23±2.35 and 32.91±1.8% at 48 and 72 h, respectively. The proliferation inhibition appeared to be time-dependent. A significant difference was observed between the MSC experimental (2.85±0.12%, 2.77±0.25%) and negative control (2.89±0.11%) groups at 24 h (P<0.01). No difference was observed between the negative and the blank control groups throughout the co-culture process.

### HSC cell cycle

**Figure 1:**
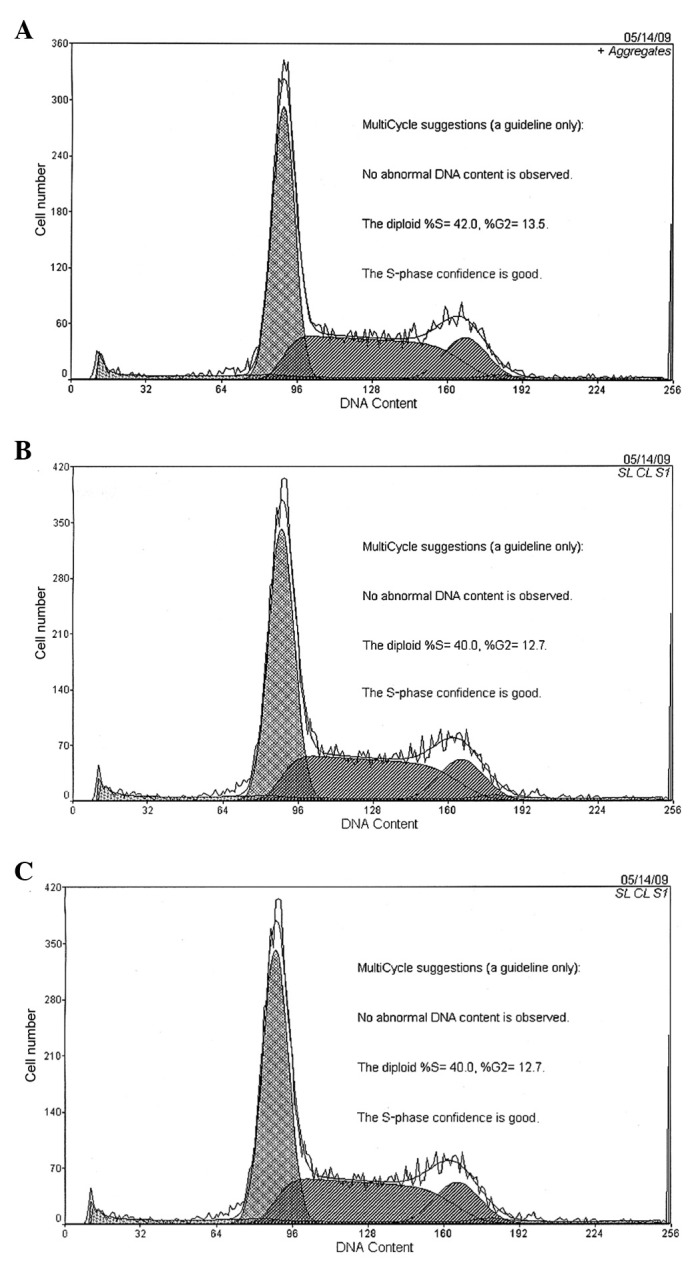
HSC cell cycle flow chart. (A) MSC experimental group prior to co-culture; (B) negative control group co-cultured for 72 h; (C) MSC experimental group co-cultured for 72 h.

Following 12 h of co-culture with MSCs, the cell number of the HSCs blocked in the G0/G1 phase increased significantly (P<0.01) in the experimental group, and the S-phase cells were significantly reduced (P<0.01) compared with the control and negative control groups. The G0/G1-phase cells were 49.45±0.95, 54.28±0.99, and 58.64±1.10%, whereas the S-phase cells were 38.86±1.17, 35.42±0.94 and 33.5±0.78% at 24, 48, and 72 h, respectively. The results revealed no difference between the negative and blank control groups throughout the co-culture process (Fig. 1).

### RhoA, cyclin D1, and P27 mRNA expression

**Figure 2:**
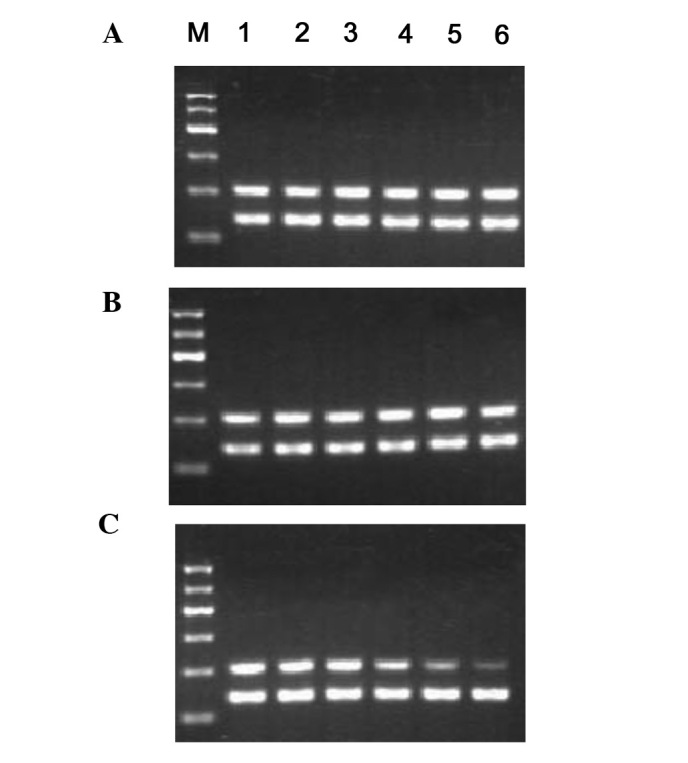
HSC RhoA mRNA expression. (A) Control group; (B) negative control group; (C) MSC experimental group. Lane 1, 0 h; lane 2, 6 h; lane 3, 12 h; lane 4, 24 h; lane 5, 48 h; lane 6, 72 h; lane M, marker.

**Figure 3:**
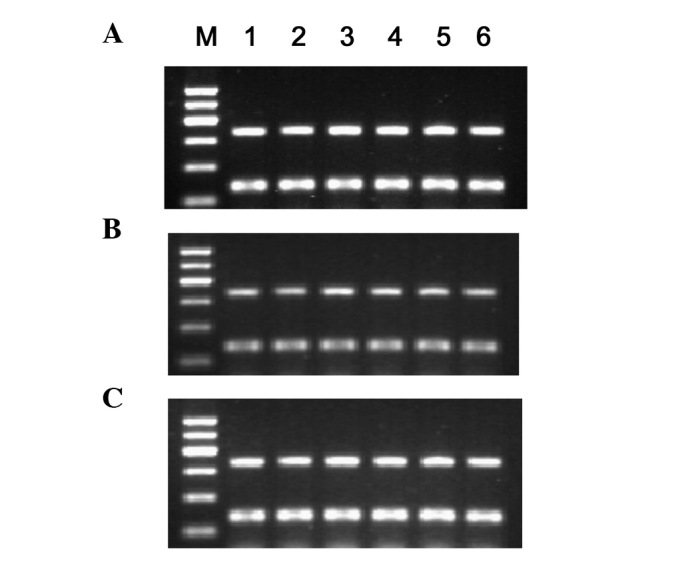
HSC p27 mRNA expression. (A) Control group; (B) negative control group; (C) MSC experimental group. Lane 1, 0 h; lane 2, 6 h; lane 3, 12 h; lane 4, 24 h; lane 5, 48 h; lane 6, 72 h; lane M, marker.

Following 12 h of co-culture, the MSC *RhoA* mRNA expression in the experimental group (0.89±0.02%) was significantly lower than that of the control group (1.06±0.02%) (P<0.01). The expression then decreased rapidly and achieved its minimum level at 72 h (0.37±0.05%). During the co-culturing period, the *RhoA* mRNA expression in the negative control (1.07±0.03, 1.03±0.05, 1.06±0.03, 1.04±0.07, 1.01±0.06 and 0.96±0.10%) and control groups (1.08±0.02, 1.04±0.03, 1.06±0.02, 0.96±0.08, 1.00±0.06 and 0.92±0.07%) exhibited no difference (Fig. 2). Following 24 h of co-culture, the *cyclin D1* mRNA expression began to decrease in the MSC group (0.71±0.03, 0.57±0.03, 0.40±0.01 and 0.28±0.02%), was markedly lower than that of the control (0.72±0.01, 0.71±0.01, 0.71±0.02, 0.70±0.02, 0.70±0.01 and 0.72±0.02%) and the negative control (0.69±0.03, 0.71±0.02, 0.70±0.01, 0.72±0.01, 0.71±0.01 and 0.70±0.02) groups at 72 h, and significant difference (P<0.01) was observed. The *P27* mRNA expression in each group showed no difference (Fig. 3) throughout co-culture period. No significant correlation (r=−0.105) between *RhoA* and *P27* mRNA expression was observed.

### RhoA, cyclin D1, and P27 protein expression

**Figure 4:**
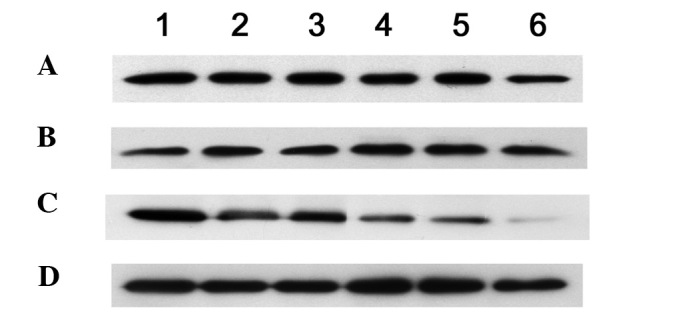
HSC RhoA protein expression. (A) control group. (B) negative control group. (C) MSC experimental group. (D) GAPDH. Lane 1, 0 h; lane 2, 6 h; lane 3, 12 h; lane 4, 24 h; lane 5, 48 h; lane 6, 72 h.

After 12 h of co-culture, RhoA protein expression (0.86±0.07%) was significantly lower in the MSC experimental group compared with that in the control group (1.11±0.12%) (P<0.01). The expression then decreased slowly and reached its lowest level at 72 h (Table I and Fig. 4).

**Figure 5:**
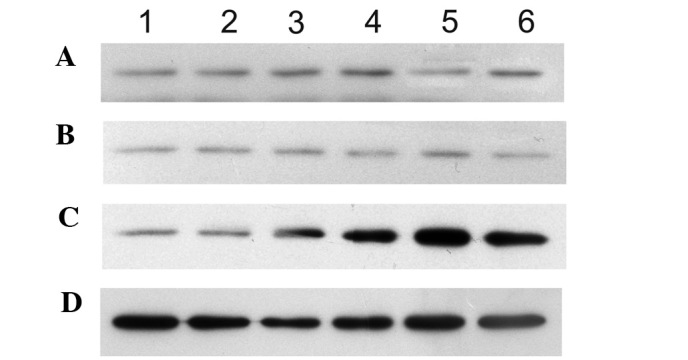
HSC p27 protein expression. (A) control group. (B) negative control group. (C) MSC experimental group. (D) GAPDH. Lane 1, 0 h; lane 2, 6 h; lane 3, 12 h; lane 4, 24 h; lane 5, 48 h; lane 6, 72 h.

After the MSCs had been co-cultured for 24 h, the cyclin D1 protein (0.65±0.09%) began to decrease, and its expression (0.11±0.06%) was significantly lower than that in the control and experimental control (P<0.01) groups at 72 h. After 12 h of co-culture, the P27 protein expression in the MSC experimental group (0.39±0.03%) increased compared with that in the control group (0.20±0.04%) (P<0.05). After 24 h of co-culture, the P27 protein (0.73±0.07%) expression significantly increased in the experimental group MSCs compared with that in the control group (0.20±0.04%) (P<0.01) and maintained high expression (Table II and Fig. 5). No difference was observed in the RhoA and P27 protein expression between the negative and blank control groups at the various co-culture time points. A significant negative correlation (r=−0.943, P<0.01) was observed in the RhoA and P27 protein expression.

## Discussion

The molecular bases of cell proliferation, achieved through the operation of the cell cycle, include cell cycle proteins (cyclin A–H), cyclin-dependent protein kinases (CDK1-7), and cyclin-dependent protein kinase inhibitors (including P21, P27 and P18). These control elements are closely linked and form a center of the cell cycle CDK regulatory network. The G0/G1-S-phase check points are regulated by G1-phase cyclin D1 (14,15).

The ability of cells to pass from the G1 to the S phase through the restriction point depends largely on cyclin D1 accumulation during the G1 phase. Cyclin D1 combines with the CDK to form complexes, conduct phosphorylation mediated by the CDK kinase and promote expression of certain genes. These gene expression products promote the passage of the cells through the G1-S regulation point and induce the cells to undergo the process of cell self-division (16). By contrast, if cyclin D1 expression is blocked, the cells cannot pass from the G1 to the S phase.

P27 is a member of the CKI family, which mainly inhibits CDK by combining with cyclin. The P27 inhibition of CDK involves two aspects: P27 inhibits cyclin CDK activity or inhibits the activation of CDK, which ultimately inhibits the cell cycle G1→S transition (17,18). In the present study, the MSCs and HSCs were cultured for 24 h. The results showed that the percentage of cells in the S phase after 24 h decreased significantly compared with that in the control group. Proliferation was significantly inhibited, cyclin D1 mRNA and cyclin D1 protein expression significantly decreased, and P27 protein increased significantly. There was a statistically significant difference between the MSC experimental and the control groups. This condition indicates that the inhibition of the proliferation of HSCs by MSCs may be through downregulation of cyclin D1 expression and upregulation of P27 protein expression. The cell cycle was arrested at the G0/G1 phase, thereby inhibiting rat HSC proliferation.

RhoA is a member of the RhoGTP kinase family, which regulates cytoskeletal dynamics, gene transcription, cell cycle progression, and cell transformation functions (19,20). Seasholtz *et al* (21) found that the RhoA activation of the PI3K pathway can be reduced by P27 protein expression and leads to changes of its own DNA synthesis, thereby regulating cell proliferation and migration. The Rho pathway inhibitor, lovastatin, or the exoenzyme C3 are capable of enhancing the efficiency of the translation of P27 mRNA. RhoA also regulates the Skp2-P27 pathway and promotes cell cycle G1/S-phase transition (22). P27 regulates cell migration through combination with RhoA to inhibit RhoA activity (23). The present study also found that, in cells cultured for 12 h, the RhoA protein expression of the HSCs was significantly reduced, whereas the P27 protein expression was significantly increased. There was a significant negative correlation between the P27 protein and the RhoA protein expression. MSCs suppressed HSC RhoA expression, and the decreased RhoA activity led to decreased P27 protein degradation. A large amount of P27 protein accumulated in the intracellular matrix, resulting in a large number of HSCs being arrested in the cell cycle in the G0/G1 phase. The cell cycle was arrested during the early period of DNA synthesis, eventually leading to HSC cell division and proliferation reduction, reduced activity, and promotion of apoptosis. The changes were significantly time-dependent.

However, the P27 mRNA expression did not change significantly in any of the co-culture groups throughout the process. P27 is not regulated at the transcriptional level (24), and P27 upregulation may be associated with the blocking of P27 degradation in the cytoplasm. The main regulation of P27 protein expression occurs in the post-translational level.

In the co-culture model, the HSC cell morphology, activity, growth inhibition rate, and protein, as well as mRNA, expression levels of RhoA and P27 did not change significantly at the 0, 6 and 12-h periods. This was probably related to the paracrine nature of the two cells or to their secretion of certain cytokines and growth factors, such as IL-10, TNF-α, GM-CSF (25), HGF (26) and NGF (27), among others. These active factors may interact and lead to changes in the microenvironment.

In conclusion, BMSCs may regulate HSCs and cyclin D1 via the RhoA-P27 pathway, which causes the cell cycle G1/S phase transition, inhibits HSC proliferation and promotes apoptosis.

## Figures and Tables

**Table I:** HSC RhoA protein/GAPDH gray ratio following co-culture (n=3, mean ± SD).

Group	Time (h)					
	0	6	12	24	48	72
Blank control	1.17±0.04	1.14±0.09	1.11±0.12	1.09±0.08	1.09±0.05	1.07±0.05
Negative control	1.07±0.16	1.03±0.25	1.06±0.16	1.06±0.17	1.05±0.28	0.99±0.27
MSCs	1.18±0.10	1.03±0.15	0.86±0.07^a^	0.60±0.11^a^	0.46±0.03^a^	0.18±0.03^a^

a P<0.01 vs. blank control.

**Table II:** HSC p27 protein/GAPDH gray ratio following co-culture (n=3, mean ± SD).

Group	Time (h)					
	0	6	12	24	48	72
Blank control	0.19±0.02	0.19±0.03	0.20±0.04	0.20±0.04	0.21±0.04	0.22±0.04
Negative control	0.22±0.03	0.22±0.05	0.21±0.04	0.20±0.02	0.21±0.02	0.22±0.03
MSCs	0.13±0.03	0.14±0.03	0.39±0.03^a^	0.73±0.07^b^	1.07±0.02^b^	0.96±0.06^b^

a P<0.05;

b P<0.01 vs. blank control.
